# High-density linkage map construction and mapping QTL for yield and yield components in autotetraploid alfalfa using RAD-seq

**DOI:** 10.1186/s12870-019-1770-6

**Published:** 2019-04-27

**Authors:** Fan Zhang, Junmei Kang, Ruicai Long, Long-Xi Yu, Zhen Wang, Zhongxiang Zhao, Tiejun Zhang, Qingchuan Yang

**Affiliations:** 1 Institute of Animal Sciences, Chinese Academy of Agricultural Sciences, Beijing, China; 2 Plant Germplasm Introduction and Testing Research, United States Department of Agriculture-Agricultural Research Service, Prosser, WA USA; 3 Cangzhou Technical College and Cangzhou Academy of Agriculture and Forestry Sciences, Cangzhou, China

**Keywords:** Alfalfa, Genetic linkage map, Yield, QTL, RAD-seq

## Abstract

**Background:**

Alfalfa (*Medicago sativa L*.) is an important forage crop grown worldwide. Alfalfa is called the “queen of forage crops” due to its high forage yield and nutritional characteristics. The aim of this study was to undertake quantitative trait loci (QTL) mapping of yield and yield-related traits in an F_1_ population of two alfalfa varieties that differ in their yield and yield-related traits.

**Results:**

We constructed a high-density linkage map using single nucleotide polymorphism (SNP) markers generated by restriction-site associated DNA sequencing (RAD-seq). The linkage map contains 4346 SNP and 119 simple sequence repeat (SSR) markers, with 32 linkage groups for each parent. The average marker distances were 3.00 and 1.32 cM, with coverages of 3455 cM and 4381 cM for paternal and maternal linkage maps, respectively. Using these maps and phenotypic data, we identified a total of 21 QTL for yield and yield components, including five for yield, five for plant height, five for branch number, and six for shoot diameter. Among them, six QTL were co-located for more than one trait. Five QTL explained more than 10% of the phenotypic variation.

**Conclusions:**

We used RAD-seq to construct a linkage map for alfalfa that greatly enhanced marker density compared to previous studies. This high-density linkage map of alfalfa is a useful reference for mapping yield-related traits. Identified yield-related loci could be used to validate their usefulness in developing markers for maker-assisted selection in breeding populations to improve yield potential in alfalfa.

## Background

Biomass yield is the most important trait for alfalfa production and increasing yield is the primary goal for improving alfalfa. Yield is a complex quantitative trait affected by the interaction of genetics and the environment (G × E). Understanding the genetic basis of yield-related traits can help increase yield in alfalfa [1]. However, cultivated alfalfa is autotetraploid and its genetic composition is complex [2]. The outcrossing nature and high heterozygosity of alfalfa increase challenges in genetic analyses. Quantitative trait loci (QTL) have been mapped to study yield-related traits in alfalfa [3–5]. However, the use of low-density genetic markers such as SSRs (simple sequence repeats) and RFLPs (restriction fragment length polymorphisms) for mapping genetic loci has limited the resolution of QTL intervals in alfalfa.

SNP markers are abundant and have high coverage in plant genomes compared to RFLP and SSR markers [6]. In alfalfa, high density genetic linkage maps have been constructed using SNP markers [7] and SNPs have also been used for genome-wide association studies (GWAS) [8–11]. SNP markers can be generated in many ways including RNA-seq, genotyping-by-sequencing (GBS), and restriction associated DNA sequencing (RAD-seq). The application of RAD-seq has been reported in many species, such as pear [12], carnation [13], and eggplant [14]. However, RAD-seq has not been used in alfalfa. Using RAD-seq to develop SNP markers and construct a high density genetic linkage map to increase resolution of QTL mapping and reduce QTL interval length [15] would be valuable for identifying causal loci to enhance traits of interest.

In the present study, we used an F_1_ population of alfalfa to map QTL for yield-related traits. Genotyping was done using RAD-seq followed by SNP calling. The resulting SNPs were used to construct a high-density linkage map. Using this map, we were able to map yield-related QTL in the alfalfa population. The ultimate objective is to identify QTL and closely linked markers that can be used for molecular breeding to improve alfalfa yield production.

## Results

### Analysis of phenotypic variation

Phenotypic data for yield, plant height, shoot diameter, and branch number were analyzed using best linear unbiased prediction (BLUP). The minimum and maximum BLUP values for yield were − 65.71 and 61.65, respectively. The range of values for F_1_ plants was wider than that of the parents, reflecting the presence of transgressive segregation (Table 1). The same was true for plant height, shoot diameter, and branch number (Table 1). The kurtosis and skewness of yield and branch number were close to zero. Broad sense heritability (H^2^) was calculated as described in a previous study [16]. The H^2^ values were 46.7, 58.4, 34.9, and 32.0% for yield, plant height, shoot diameter, and branch number, respectively (Table 1). Analysis of variance (ANOVA) for yield, plant height, shoot diameter, and branch number were conducted using PROC GLM (SAS Institute, 2010). Genotypic variations were significant for yield, plant height, shoot diameter, and branch number (*p* < 0.001) when the mixed model of genotype*years*location was used (Table 2). Significant differences were also found for all traits analyzed when genotype*year and genotype*location models were used (Table 2). Correlation analysis showed a significant correlation according to Pearson’s test (*P* < 0.01) among four yield-related traits (Table 3). The correlation coefficient between yield and plant height is 0.79. The correlation coefficient between yield and shoot diameter is 0.51. The correlation coefficient between yield and branch number is 0.82 (Table 3).

**Table 1:** Phenotypic variation of BLUP value in F1 population. SE: standard error

Trait	Parent1	Parent2	Min	Max	Mean	SE	Kurtosis	Skewness	H^2^(%)
Yield	−30.77	25.52	−65.71	61.65	0.25	2.12	−0.28	−0.28	46.7
Plant height	−0.04	2.39	−9.73	7.84	0.02	0.23	1.46	−0.84	58.4
Shoot diameter	0.03	−0.03	−0.16	0.09	−0.01	0.01	13.04	1.26	34.9
Branch number	−25.31	13.60	−33.24	43.10	0.25	1.21	0.02	0.07	32.0

**Table 2:** Analysis of variance for yield, plant height, shoot diameter and branch number in a F1 population in 3 years at two location, Langfang and Tongzhou (5 environments) using mixed model

	df	Type III SS	*F* value	*P* value
Yield				
genotype	151	3,762,939.34	4.41	<.0001
years	2	3,824,983.93	338.66	<.0001
location	1	10,911,768.51	1932.22	<.0001
replication	2	1,668,561.23	147.73	<.0001
years*location	1	2,218,982.40	392.93	<.0001
genotype*years	297	2,963,096.86	1.77	<.0001
genotype*location	150	1,751,503.34	2.07	<.0001
genotype*years*location	135	1,184,647.91	1.55	0.0001
Plant height				
genotype	151	161,263,035.90	13.79	<.0001
years	2	254,959.70	1.65	0.1933
location	1	3,665,115.00	47.31	<.0001
replication	2	507,343.50	3.27	0.0381
years*location	1	176,871.10	2.28	0.131
genotype*years	297	38,969,640.00	1.69	<.0001
genotype*location	150	51,820,222.70	4.46	<.0001
genotype*years*location	135	46,567,880.30	4.45	<.0001
Shoot diameter				
genotype	151	7,939,652.33	6.82	<.0001
years	2	302,227.26	19.59	<.0001
location	1	12,854.73	1.67	0.1969
replication	2	4805.17	0.31	0.7324
years*location	1	57,332.74	7.43	0.0065
genotype*years	291	7,345,673.35	3.27	<.0001
genotype*location	148	3,409,196.28	2.99	<.0001
genotype*years*location	123	5,789,746.50	6.10	<.0001
Branch number				
genotype	151	3,511,180.98	4.69	<.0001
years	2	380,363.09	38.34	<.0001
location	1	565,387.34	113.99	<.0001
replication	2	13,300.68	1.34	0.262
years*location	1	15,739.49	3.17	0.0751
genotype*years	297	2,395,604.91	1.63	<.0001
genotype*location	150	2,896,988.25	3.89	<.0001
genotype*years*location	135	2,557,434.42	3.82	<.0001

**Table 3:** Correlation coefficient matrix for yield related traits

	Yield	Plant height	Shoot diameter	Branch number
Yield	1.00			
Plant height	0.79	1.00		
Shoot diameter	0.51	0.64	1.00	
Branch number	0.82	0.58	0.22	1.00

### Genetic linkage map

**Fig. 1:**
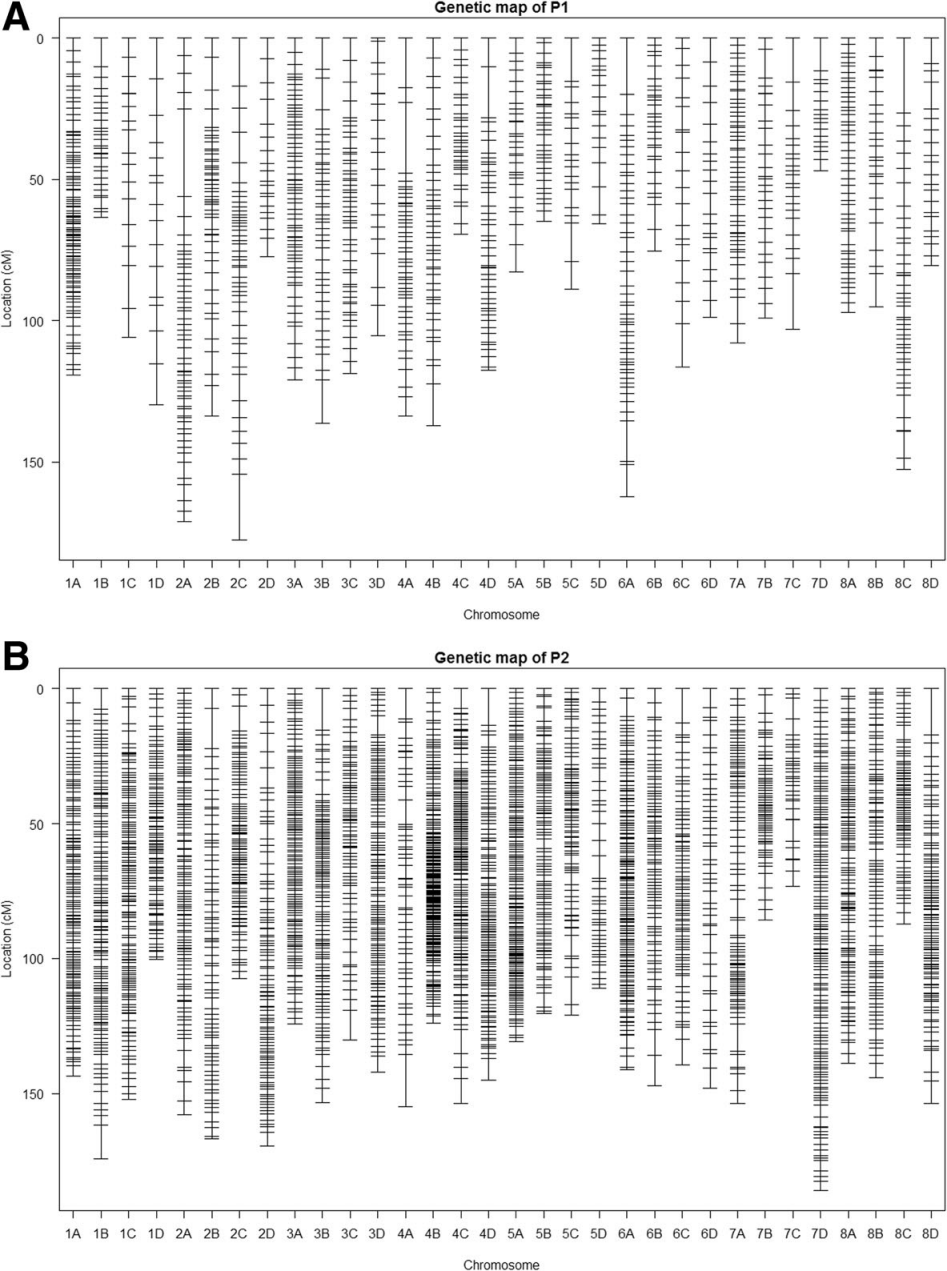
Genetic map showing 32 linkage groups (LGs) for the P1(**a**) and P2(**b**) of the mapping population. The positions of markers are showed in Kosambi centimogan(cM). Each chromosome is named based on *Medicago truncatula*, with four LGs (A to D) on each of eight chromosomes

A total of 113,837 SNP markers were obtained after genotype calling with the Universal Network Enable Analysis Kit (UNEAK) pipeline. After removal of markers with missing values > 50%, 82,668 SNP markers were retained. Of those, 2317 were single-dose allele (SDA) markers in P1 and 4553 SDA in P2. For SSR markers, 56 and 84 SDA markers were obtained in P1 and P2, respectively. The SNP markers and SSR markers were combined to construct linkage maps. Thirty-two linkage groups were obtained for each parent (Fig. 1). The chromosome numbers were assigned based on SSR markers with known chromosome positions and the alignment of SNP markers sequences to the physical map of *Medicago truncatula*.

The P1 linkage map spanned a total of 3455 cM with 1153 mapped markers and an average marker density of 3.00 cM. The highest number of markers were found in linkage group (LG) 1A (106 markers) and lowest in LGs 1C and 1D (16 markers in each LG) (Table 4). The P2 linkage map spanned a total of 4381 cM with 3312 mapped markers and an average marker density of 1.32 cM (Fig. 1a and Table 4). The highest number of markers was found in LG 4B (201 markers) and the lowest in LG 7C (31 markers) (Fig. 1b, Table 4).

**Table 4:** Distribution of SNP and SSR markers among 32 linkage groups of P1 and P2 parents of the F1 mapping population and the length of each haplotype map

Chromosome	Haplotype	P1		P2	
		Marker, No.	Length, cM	Marker, No.	Length, cM
1	A	106	119.29	134	143.57
1	B	28	63.51	134	174.20
1	C	16	105.78	129	152.18
1	D	16	129.92	98	100.46
2	A	56	171.09	114	157.76
2	B	51	133.74	71	166.88
2	C	45	177.57	94	107.61
2	D	19	77.44	94	169.50
3	A	65	120.89	126	124.26
3	B	41	136.23	110	153.46
3	C	49	118.70	75	130.09
3	D	19	105.31	118	142.12
4	A	46	133.83	52	154.89
4	B	43	137.31	201	123.92
4	C	33	69.39	158	153.81
4	D	44	117.67	120	144.94
5	A	31	82.95	172	130.78
5	B	36	64.88	101	120.26
5	C	19	88.91	84	120.89
5	D	18	65.83	46	110.99
6	A	50	162.30	156	141.20
6	B	29	75.32	83	147.24
6	C	20	116.54	80	139.25
6	D	21	98.77	47	147.95
7	A	51	107.81	91	153.73
7	B	26	98.98	62	85.89
7	C	22	103.03	31	73.33
7	D	17	46.90	143	185.75
8	A	48	96.98	113	138.81
8	B	25	94.93	93	144.17
8	C	42	152.75	77	87.31
8	D	21	80.46	105	153.82
Total		1153	3455.01	3312	4381.02
average			3.00		1.32

### QTL mapping

**Fig. 2:**
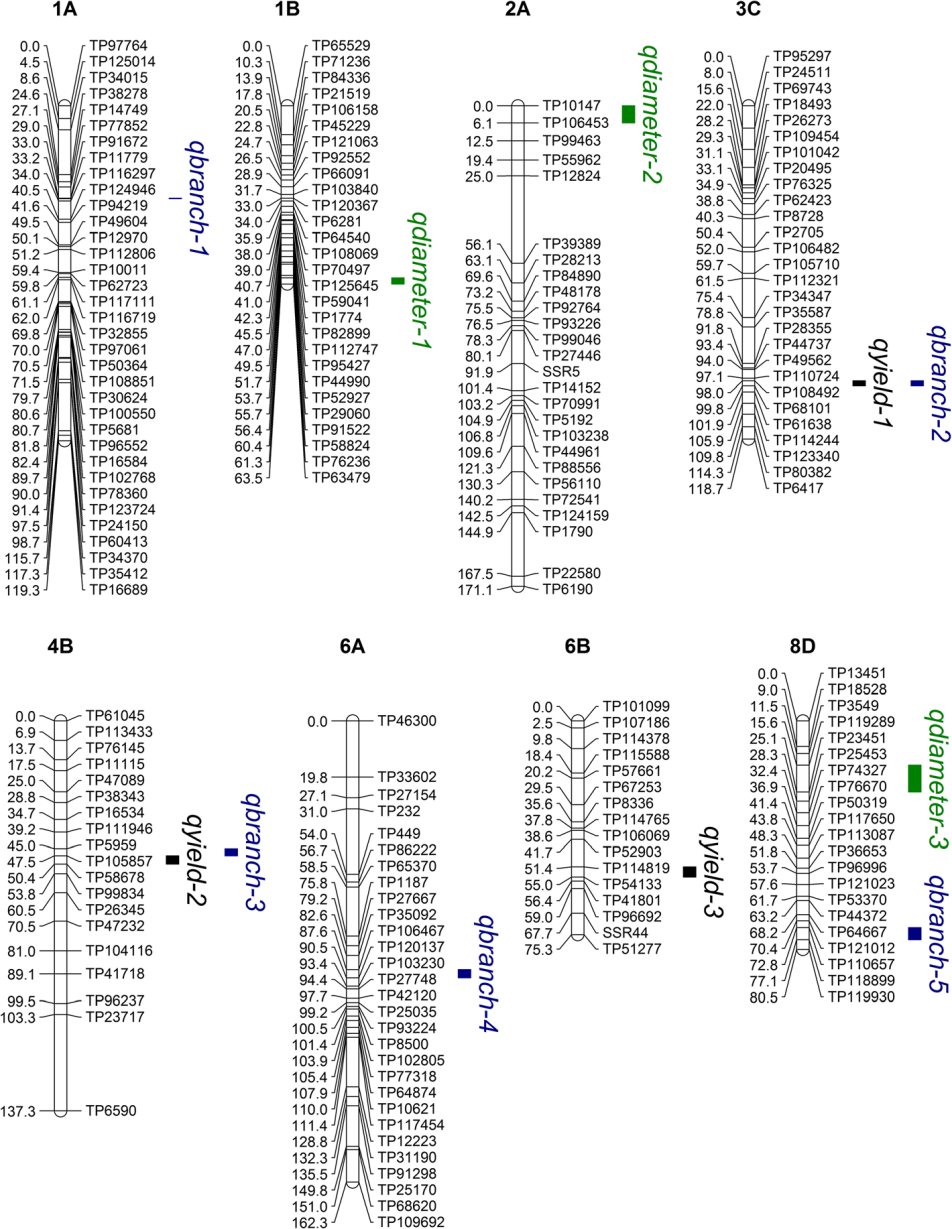
Yield related QTL on 32 linkage groups from a genetic linkage map of parent 1. Each linkage group is named based on *Medicago truncatula*, with four group ordered A to D randomly. The name *qyield, qheight, qdiameter and qbranch* represent the QTL of yield, plant height, shoot diameter and branch number using blup value. Distances among markers are indicated in cM to the left of the linkage groups; names of markers are shown on the right. Only those SNP markers are shown which were in and around the QTL regions. QTLs are depicted as colored vertical bars to the right of the linkage groups. Different colors represent different traits. Those groups have not detected QTL are not been shown

**Fig. 3:**
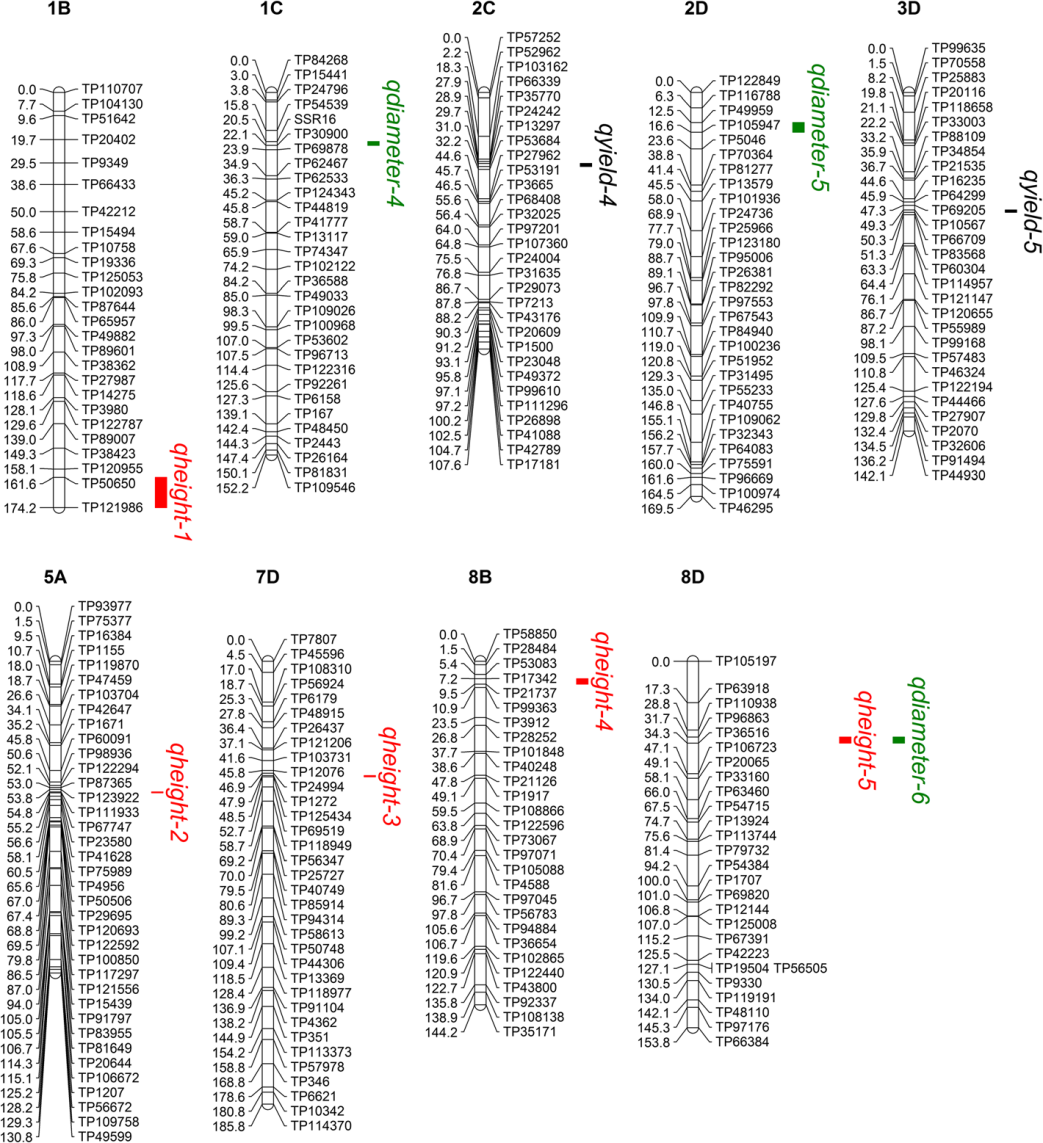
Yield related QTL on 32 linkage groups from a genetic linkage map of parent 2. Each linkage group is named based on *Medicago truncatula*, with four group orderd A to D randomly. The name *qyield, qheight, qdiameter and qbranch* represent the QTL of yield, plant height, shoot diameter and branch number using BLU*P* values. Distances among markers are indicated in cM to the left of the linkage groups; names of markers are shown on the right. Only those SNP markers are shown which were in and around the QTL regions. QTLs are depicted as colored vertical bars to the right of the linkage groups. Different colors represent different traits. Those groups have not detected QTL are not been shown

Phenotypic data was collected from five environments: 3 years from Langfang (LF2014, LF2015, LF2016) and 2 years from Taizhou (TZ2014, TZ2015) were used for QTL mapping. Phenotypic data were analyzed using BLUP and the BLUP values were used for QTL mapping. A total of 21 QTL were identified, including five QTL for yield, five QTL for plant height, five QTL for branch number, and six QTL for shoot diameter in two parents (Table 5). Five QTL explained more than 10% of the phenotypic variation (PVE). The PVE ranged from 4.4 to 13.6% for yield related traits. Among those, three pairs of QTL were co-located: *qyield-1* and *qbranch-2*, *qyield-2* and *qbranch-3*, and *qheight-5* and *qdiameter-6* (Figs. 2 and 3). Sequences and SNP variance of the nearest markers of every QTL were supplied (Table 6).

**Table 5:** Quantitative trait loci (QTL) associated with yield related traits identified by inclusive composite interval mapping of additive effects. Trait names, QTL, linkage group (LG), LG position, the 1-LOD support interval in cM unit (LOD interval), the logarithm of the odds (LOD), the percentage of the phenotypic variation explained by QTL, the additive effects (Add) of the QTL are presented

Parent	Trait name	QTL	LG	Position (cM)	LOD interval (cM)	Flanking markers	LOD	PVE(%)	Add
P1	yield	*qyield-1*	3C	98.0	98.0–99.8	TP108492-TP68101	4.3	13.6	13.8
P1	yield	*qyield-2*	4B	45.5	45.0–47.5	TP5959-TP105857	5.5	8.6	11.3
P1	yield	*qyield-3*	6B	52.0	51.4–55.0	TP114819-TP54133	5.9	8.9	11.2
P2	yield	*qyield-4*	2C	30.5	29.7–31.0	TP24242-TP13297	4.6	7.2	9.9
P2	yield	*qyield-5*	3D	50.0	49.3–50.3	TP10567-TP66709	3.0	7.3	−9.9
P2	plant height	*qheight-1*	1B	163.5	161.6–174.2	TP50650-TP121986	3.1	9.8	−1.1
P2	plant height	*qheight-2*	5A	55.0	54.8–55.2	TP111933-TP67747	4.3	8.3	1.0
P2	plant height	*qheight-3*	7D	48.0	47.9–48.5	TP1272-TP125434	3.9	7.3	1.0
P2	plant height	*qheight-4*	8B	7.5	7.2–9.5	TP17342-TP21737	3.8	6.6	−0.9
P2	plant height	*qheight-5*	8D	32.5	31.7–34.3	TP96863-TP36516	4.3	6.1	0.9
P1	shoot diameter	*qdiameter-1*	1B	62.5	61.3–63.5	TP76236-TP63479	5.3	12.2	0.0
P1	shoot diameter	*qdiameter-2*	2A	3.0	0.0–6.1	TP10147-TP106453	3.4	7.3	0.0
P1	shoot diameter	*qdiameter-3*	8D	16.0	15.6–25.1	TP119289-TP23451	3.8	8.7	0.0
P2	shoot diameter	*qdiameter-4*	1C	21.0	20.5–22.1	SSR16-TP30900	3.9	7.1	0.0
P2	shoot diameter	*qdiameter-5*	2D	13.0	12.5–16.6	TP49959-TP105947	6.9	13.6	0.0
P2	shoot diameter	*qdiameter-6*	8D	32.0	31.7–34.3	TP96863-TP36516	4.0	9.0	0.0
P1	branch number	*qbranch-1*	1A	33.0	33.0–33.2	TP91672-TP11779	3.2	7.4	5.1
P1	branch number	*qbranch-2*	3C	98.5	98.0–99.8	TP108492-TP68101	4.3	13.5	6.8
P1	branch number	*qbranch-3*	4B	48.0	47.5–50.4	TP105857-TP58678	3.6	4.4	4.1
P1	branch number	*qbranch-4*	6A	88.0	87.6–90.5	TP106467-TP120137	9.0	12.1	6.6
P1	branch number	*qbranch-5*	8D	74.5	72.8–77.1	TP110657-TP118899	3.9	8.0	−5.6

**Table 6:** Sequences and SNP variance of the nearest markers of every QTL

QTL	Nearest marker	Variants	Sequences/primer sequences
*qyield-1*	TP108492	G/T	AATTCTGCAGGAAATCCCTGGGTTTTTAGGAACTCGTGGCTGATTGTGAAGCCTTGAGAGACGA
*qyield-2*	TP5959	A/T	AATTCAAATCAATCAAAGCAAAGATGTAGTATATGTTCACCAAACAAAATACACAAAGGAGCTT
*qyield-3*	TP114819	A/G	AATTCTTATATGATGTAAATAATGAGGAATTGAAATTGAGGGTGTTTTTATGGAAATATAATTA
*qyield-4*	TP13297	A/G	AATTCAAGGTTACAGTCAATTGTAGAAGCCAAAAGCTACAAATTCTAGCTTCAAGTAAAATCAA
*qyield-5*	TP66709	A/C	AATTCCTCGTAGTCGCTACATTAAATCCTCTCTATGGTCTGGCATTAAGGATCATATGAATATG
*qheight-1*	TP50650	A/C	AATTCCAACATGCATAAATACTCAAAAACAGCAAAGTACAAAAAAAATAAAGGGACAATCAAGT
*qheight-2*	TP67747	A/T	AATTCCTGGAAATATGTCCCCACCAACTAAGAGCAAAGGAAAAGAAAATTAAGGCAAAACATCA
*qheight-3*	TP1272	A/T	AATTCAAAACTTTGGTTAAATTGCTAAGTGGTCCCTAAGCATCCACATGCTATGTATTGGATTA
*qheight-4*	TP17342	G/T	AATTCAATGTATATGAATATTGTATCTTCGCATTAAAATGATATCCATGCATTGGAACAGCGAC
*qheight-5*	TP96863	A/T	AATTCTATAGACAAATTAGTTTAAATTCAAAATACATGACGAGATTTGTTTCATATGTGTTAGT
*qdiameter-1*	TP63479	C/T	AATTCCGTAATGGCGTCAAGTAACCCTTCGCTTCGCCCTGAGATCGGACCCGACGGTCTTGCCA
*qdiameter-2*	TP10147	A/G	AATTCTCATTATTGCCAATATCCTTCCACTGTAGCCGCCAATTTTTGTATTTTTTTTTTTTAAT
*qdiameter-3*	TP119289	A/G	AATTCTTGAGTTGGAGAAGCACACCATAATTTCTATATATTTATGTTGCACACCACAATTGAAG
*qdiameter-4*	SSR16	–	(forward) CACCACTATCTCTTCCCTCACC/ (reverse) TGTTGGTAATGTTCAAGCTCCA
*qdiameter-5*	TP49959	A/C	AATTCCAAAGCTTTGGGGAGGCTAAGGTCAGCATGTGAGAAAGCAAAGAGGTTACCTTCTTCAA
*qdiameter-6*	TP96863	A/T	AATTCTATAGACAAATTAGTTTAAATTCAAAATACATGACGAGATTTGTTTCATATGTGTTAGT
*qbranch-1*	TP91672	G/T	AATTCTAATAATACTTAGACTTTCTTTTGCAAAAGGACTAAATCAACCTCACACTTTCACAATC
*qbranch-2*	TP108492	G/T	AATTCTGCAGGAAATCCCTGGGTTTTTAGGAACTCGTGGCTGATTGTGAAGCCTTGAGAGACGA
*qbranch-3*	TP105857	C/T	AATTCTCTTGAAGAGGGAGAGTATGATGTGATCCTAAAATTCTATTTCTTGACAAGAATAATAA
*qbranch-4*	TP106467	A/T	AATTCTGAAACACCATGGCCTGGAGACGATAGAGCTTGCACTAGAGTAGAAGAGTCGGTCTCGA
*qbranch-5*	TP110657	A/T	AATTCTGGGTTTGCAATAATAATGAAAAAGAAGATTAAAGTAACAAAGAAAGAACCTTGTCCAT

### Yield

QTL analysis identified a total of five QTL for yield. The phenotypic variation explained (PVE) by a single QTL ranged from 7.2 to 13.6%. The highest value of PVE, 13.6%, was found for the QTL *qyield-1*. It was located on linkage group 3C of P1 at the position from 98.0 to 99.8 cM with a logarithm of odds (LOD) value of 4.3 (Table 5). The QTL *qyield-1* overlapped with the reported QTL for forage yield (72.3–102.2 cM) [3] and root dry weight (93–117 cM) [17]. Similarly, the other four QTL (*qyield-2*, *qyield-3*, *qyield-4*, and *qyield-5*) were also detected within a similar position as QTL for yield. The QTL *qyield-2* was in a similar location as other reported QTL for forage yield (43.8–54.5 cM) [3] and root dry weight (39–55 cM) [17]. The QTL *qyield-2* identified in the present study was also co-located with the reported QTL for fall plant height QTL [17] and seeds per seedpod QTL [18]. The QTL *qyield-3* was located within the reported QTL for shoot dry weight and winter injury [17]. The QTL *qyield-4* was located at a similar position as the reported QTL for crown dry weight (8–30 cM) and fall plant height (24–40 cM) [17]. The QTL *qyield-5* was in a similar location as reported QTL for forage yield (42.3–48.8 cM) and winter hardiness (47.6–50.1 cM) [19].

### Plant height

Five QTL with PVE ranging from 6.1 to 9.8% were identified for plant height. They were mapped to five LGs (1B, 5A, 7D, 8B, 8D). All of these QTL were identified in the high yield parent (P2). However, contrary to QTL *qheight-2*, *qheight-3*, and *qheight-5*, QTL *qheight-1* and *qheight-4* had negative effects (Add< 0) on plant height (Table 5). There were two QTL (*qheight-2* and *qheight-3*) mapped within similar positions of reported QTL for plant height in alfalfa. The QTL *qheight-2* identified in this study was located at a similar position as the reported QTL for fall plant height (48–68 cM), shoot dry weight (50–70 cM), and root dry weight (52–70 cM) [17]. The QTL *qheight-3* was located within a QTL for fall plant height identified in a previous study [17]. Additional reports of QTL for fall dormancy (47.3–52 cM) [19] were mapped in the same chromosome region as *qheight-3*. Furthermore, *qheight-4* was located within the genomic location of shoot dry weight (0–17 cM) and root dry weight (0–27 cM) [17].

### Shoot diameter

For shoot diameter, six QTL were identified, explaining 7.1 to 13.6% of the phenotypic variance. The LOD value ranged from 3.4 to 6.9. The QTL *qdiameter-5* on LG 2D (position 12.5–16.6 cM) had the highest LOD of 6.9 and highest PVE of 13.6% (Table 5). The QTL *qdiameter-5* was located in a similar location as previously reported QTL for crown dry weight (8–30 cM) in alfalfa [17]. Similarly, three other QTL (*qdiameter-1*, *qdiameter-2*, and *qdiameter-3*) were located in a similar position as yield-related traits. The QTL *qdiameter-1* was in a location similar to QTL for root dry weight (60–70 cM) [17] and drought-stressed forage biomass (59–72 cM) [20]. The QTL *qdiameter-2* was located in a similar position as reported QTL for shoot dry weight (0–8 cM) [17]. The QTL *qdiameter-3* was in a similar location as reported QTL for shoot dry weight (0–23 cM), crown dry weight, and root dry weight (17–29 cM) [17]. The QTL *qdiameter-4* was in a similar location as reported QTL for fall dormancy (21.1–23.7 cM) [19].

### Branch number

Among the five QTL identified for branch number, two QTL (*qbranch-2* and *qbranch-4*) had PVE greater than 10%. The QTL *qbranch-2* was located on linkage group 3C of P1 at the position from 98.0 to 99.8 cM. The PVE of *qbranch-2* was 13.5%. The QTL *qbranch-4* was located on linkage group 6A of P1 at the position from 87.6 to 90.5 cM. The PVE of *qbranch-4* was 12.1% (Table 5). The QTL *qbranch-2* was located in a similar genomic location of reported QTL for root dry weight (93–117 cM) and shoot dry weight (101–127 cM). The QTL *qbranch-4* was in a similar location as reported QTL for fall plant height (82–101 cM) [17]. Both of these QTL had positive effects (Add> 0) for branch number.

## Discussion

### The use of RAD-seq and methods for allele calling

RAD-seq has been used to generate SNP markers in many plant species, including barley [21], pear [12], and grape [22]. It has been applied to construct high-density genetic linkage maps in plant species [22], however, RAD-seq has not been used in alfalfa. Both GBS and RAD-seq use methylation-sensitive enzymes for cutting genome sequences to reduce redundancy. However, the sensitive enzymes and sequencing strategies are different for the two method [23]. GBS has been used for generating SNPs in alfalfa [7, 19]. We are the first to use RAD-seq to generate SNPs to construct high density linkage maps in alfalfa with improved coverage. RAD-seq used in the present study produced more SNP markers (113,837) compared to previous studies using GBS [7, 19].

In the present study, we used the UNEAK pipeline for SNP calling. UNEAK was developed for GBS analysis in species with non-reference genomes. We used the UNEAK pipeline and called SNPs with RAD-seq. The combination of RAD-seq and the UNEAK pipeline provides a comprehensive strategy to obtain an adequate number of markers for QTL mapping. We compared the UNEAK pipeline with the reference pipeline, TASSEL-GBS [24], for genotype calling. The UNEAK pipeline gave better results for genotype calling than the reference pipeline. Using the UNEAK pipeline, we obtained high density SNPs and constructed linkage maps with 32 linkage groups (LGs). In contrast, we were unable to group the variants called using the reference pipeline and could not establish 32 LGs. This was probably because the reference genome of *M. truncatula* was diploid and did not align well with the tetraploid alfalfa genome, resulting in losing genetic information of alfalfa when aligned to the reference genome. The UNEAK pipeline is a platform using network comparison without alignment to the reference genome, and therefore, it resulted in more markers than the reference pipeline.

### Marker density

The marker density is comparable to that of the genetic linkage map in alfalfa constructed in previous studies [7, 19]. This congruence suggests that the genotyping method used in this study is appropriate for a genetic mapping approach in alfalfa. However, the total length of the P1 map (3455 cM) was less than that of the P2 map (4381 cM). Different coverages were also found between parents in the previous studies [7, 19, 25]. Genetic differences in the parents are likely the main reason for different SNP coverage between the parents [26]. Furthermore, sequencing bias may cause a low number of raw reads for P1. Nevertheless, we were able to map 3312 markers to 32 LGs that covered all four sets of genomes in tetraploid alfalfa.

QTL for yield-related traits in alfalfa have been reported in previous studies [5, 17, 27]. However, the genetic maps were constructed using SSR and RFLP markers [5, 27] and the limited coverage provided by SSR and RFLP platforms resulted in large QTL intervals (> 10 cM) with low resolution. The use of single-dose alleles (SDAs) for genetic mapping is feasible in tetraploid species. Adhikari et al. identified QTL for fall dormancy and winter hardiness in alfalfa F_1_ population using SDAs [19]. This method is a pseudo-testcross strategy, which uses the simplex markers (AAAB x BBBB) of an F_1_ population for autotetraploid genetic linkage map construction using diploid software like JoinMap [28]. The pseudo-testcross strategy allows us to use thousands of SDA markers to construct linkage map followed by QTL mapping. Using SDA markers and the composite interval mapping method, QTL intervals were greatly reduced (< 3 cM) in the present analysis.

### Comparison of QTL associated with yield-related traits in alfalfa

To reduce the effect of the interaction between genetics and the environment (G × E), we used BLUP to estimate phenotypic variation across five environments and identified QTL for each trait analyzed in this study. Among 21 QTL associated with yield and yield-related traits, several QTL were co-located among the yield-related traits. This indicated that phenotyping in multiple environments and adjusted BLUP is a useful way to control environmental variation. Most QTL identified in the present study were co-located with previously reported yield-related QTL [3, 17] and fall dormancy QTL [19]. There were five QTL (*qyield-1*, *qbranch-2*, *qbranch-4*, *qdiameter-1*, and *qdiameter-5*) with PVE > 10% and all of them are co-located with reported yield QTL except *qbranch-4*. These QTL may play a major role in controlling yield. Additional QTL (such as *qyield-2* and *qheight-2*) were co-located with other traits, such as fall dormancy QTL, which may suggest pleiotropic effects of the genes.

We are the first to map QTL for shoot diameter and branch number in alfalfa. Although the heritability was not very high (34.9 and 32.0% for shoot diameter and branch number, respectively), we were able to detect six QTL for shoot diameter and five QTL for branch number. Several QTL for shoot diameter and branch number were co-located with QTL for yield and plant height: *qyield-1* was co-located with *qbranch-2*; *qyield-2* with *qbranch-3*; and *qheight-5* with *qdiameter-6* (Figs. 2 and 3). Furthermore, most shoot diameter and branch number QTL identified in the present study were co-located with shoot dry weight, crown dry weight, or root dry weight QTL [17]. These QTL may have an indirect effect in controlling yield traits [29].

Given that the average heritability (43%) of yield-related traits was reasonable for genetic analysis, the QTL identified in the present analysis need further validation. Overlapping QTL have been reported using different populations with different genetic backgrounds in different environments [3, 17, 19, 20]. In the present study, six QTL overlapped with three reported QTL. Among those, one of them had high PVE (> 13%). QTL with high PVE may play major roles for yield-related traits. Furthermore, in the present analysis, we were able to narrow down the QTL interval with high PVE (*qyield-1* and *qbranch-2*) to 1.8 cM (98.0–99.8 cM), which will facilitate further investigations such as fine mapping and gene cloning.

## Conclusions

In the present study, we constructed high density genetic maps using SNPs generated by RAD-seq in an F_1_ population derived from two local alfalfa cultivars Cangzhou and Zhongmu No. 1 with low and high yield, respectively. Our results showed that RAD-seq is an appropriate method for generating genetic markers that can be used to construct linkage maps in alfalfa. The QTL detected in this study will help us to understand the genetic basis of yield-related traits. However, these QTL may be not robust in different populations or environments and thus must be validated in breeding populations in future studies. With further investigation, markers closely linked to the major QTL can be used for marker-assisted selection to improve yield in alfalfa.

## Methods

### Plant materials and experimental design

P1 (paternal parent; Cangzhou) and P2 (maternal parent; Zhongmu No. 1) were individually selected from a panel of, respectively, low forage yield of Cangzhou and high forage yield of Zhongmu No. 1. They were crossed to generate an F_1_ population consisting of 149 progeny lines. In 2012, the seeds of the F_1_ population were planted in the greenhouse of the Chinese Academy of Agricultural Sciences (CAAS) in Langfang, Hebei Province, China, under conditions of 16 h day/8 h night, 22 °C and 40% relative humidity. Light intensity (a combination of natural light and incandescent lamps) was approximately 200 μmol m^− 2^ s^− 1^. Clones were propagated from individual plants by stem cuttings. During the early branching stage in 2013, the cloned plants were moved from propagation flats to the field of the CAAS research station in Langfang, Hebei Province (39.59°N, 116.59°E). F_1_ and parent individuals were also transplanted to establish a field trial in Tongzhou, Beijing (39.92°N, 116.65°E).

The field trial was carried out using a randomized complete block design (RCBD) with three replications at each of the two field sites. Every replication had one clone plant for every individual. The plant spacing was 100 cm between rows and 80 cm within rows. The individuals were not similar with each other after transplanting. Alfalfa is a perennial plant and is harvested by cutting; its rapid regrowth after cutting makes alfalfa a high yielding forage crop. To ensure that the plants were uniform before regrowth, they were clipped to a height of 5 cm; plants were clipped twice before regrowth for phenotyping. Weeds were removed manually and there was no cover crop in the field.

### Phenotypic data collection and analysis

Phenotypic data were collected at two locations in multiple years. Two and 3 years of phenotypic data were respectively collected in Tongzhou in 2014 and 2015 (TZ2014, TZ2015) and in Langfang in 2014, 2015, and 2016 (LF2014, LF2015 and LF2016). Alfalfa plants were harvested at the early flowering stage (when 10% of plants begin flowering). Yield was measured after harvested plants were dried in a forced-air dryer. Plant height was measured based on the tallest stem at the date of harvesting. Branch number (the number of all stems) was counted at the same time. Diameters of five randomly selected shoots were measured at the shoot bottom after harvesting and the mean value was calculated. Data of yield-related traits from the first harvest was used for our analysis.

Best linear unbiased predictions (BLUPs) were used for statistical analysis of phenotypic data collected from years at different locations using PROC MIXED [30]. Analyses of the interaction of genotype with location and years were conducted using PROC GLM (SAS Institute, 2010). The correlation analysis of yield-related traits were conducted using PROC CORR (SAS Institute, 2010).

The random effect model used for BLUP was as follows:

$$ {\mathrm{Y}}_{\mathrm{i}\mathrm{jkh}}=\mathrm{m}+{l}_k+{\mathrm{r}}_{\mathrm{i}\left(\mathrm{k}\right)}+{\mathrm{g}}_{\mathrm{j}}+{\mathrm{y}}_{\mathrm{h}}+{\mathrm{g}\mathrm{l}}_{\mathrm{j}\mathrm{k}}+{\mathrm{g}\mathrm{y}}_{\mathrm{j}\mathrm{h}}+{\mathrm{g}\mathrm{l}\mathrm{y}}_{\mathrm{j}\mathrm{k}\mathrm{h}}+{\mathrm{e}}_{\mathrm{i}\mathrm{jkh}} $$

where *Y*_*ijkh*_ is the Y for the *j*th genotype in the *i*th replication of the *k*th location in the *h*th year; m is the grand mean; *r*_*i(k)*_ is the effect of the *i*th replication nested in the *k*th location; *y*_*h*_ is the effect of the *h*th year; *g*_*j*_ is the genetic effect of the *j*th genotype; *gl*_*jk*_ is the interaction effect of the *j*th genotype and *k*th location; *gy*_*jh*_ is the interaction effect of the *j*th genotype and *h*th year; *gly*_*jkh*_ is the interaction effect of the *j*th genotype, *k*th location, and *h*th year; and *e*_*ijkh*_ is the residual. All factors were random effects except for the grand mean.

### DNA isolation and RAD library construction

DNA was extracted from 100 mg fresh young leaf tissue using the CWBIO Plant Genomic DNA Kit (CoWin Biosciences, Beijing, China), according to the manufacturer’s protocol. DNA was quantified using a Nanodrop 2000 spectrophotometer based on absorbance of 260 nm (Thermo Scientific, Waltham, MA, USA). DNA concentrations were adjusted to 50 ng/μl and subsequently used for RAD library preparation. DNA was digested by the EcoRI restriction enzyme (New England BioLabs, NEB), then ligated to a unique barcode adapter. Samples were pooled together and randomly sheared ultrasonically. The average length of sheared fragments was confined to around 400 bp using Qseq100 DNA Analyzer (Bioptic Inc.). The AMPure XP Beads PCR Purification kit (Beckman Coulter, Inc.) was applied to purify the sheared DNA fragments. The fragment ends were repaired with the Quick Blunting kit (NEB). A 3′-dA overhang was added using the dA-tailing module contained in the kits (NEB), and then ligated to a common adapter. The collected fragments were enriched by PCR amplification and purified using the AMPure XP Beads PCR purification kit (Beckman Coulter, Inc.). Each sample was normalized to 10 nM for sequencing using a Hi-seq X Ten (Illumina) at ORI-GENE (Beijing, China). The RAD sequences were submitted to the NCBI Sequence Read Archive with BioProject ID: PRJNA503672.

### Sequence analysis and SNP genotyping

The Tassel 3.0 Universal Network Enable Analysis Kit (UNEAK) pipeline [31] was used for SNP genotype calling. We initially used the *Medicago truncatula* genome (Mt4.0 v1) as a reference for SNP calling (TASSEL-GBS), but we could not identify linkage groups using the resulting SNP markers. Briefly, a minimum of five was used for the tag number count step for the UQseqToTagCountPlugin command line of the UNEAK build in TASSEL and a 64 bp target length was used for trimming sequences. Marker missing values were less than 50%. Other parameters were set as default.

### SSR marker genotyping

In total, 184 SSR markers were also used for genotyping. Primer sequences of SSR markers were obtained from the Samuel Roberts Noble Foundation (Ardmore, OK, USA). Polymerase chain reaction (PCR) was based on the method of Diwan et al. [32]. Briefly, PCR steps were as follows: 94 °C for 3 min, followed by 33 cycles with 30 s at 94 °C, 30 s at 56 °C, and 60 s at 72 °C, followed by an elongation step of 7 min at 72 °C. PCR products of SSR markers were separated using polyacrylamide gel electrophoresis (PAGE) and each allele of an SSR marker was scored as 1 for present, 0 for absent, and 9 for missing.

### Linkage mapping and QTL analysis

Single-dose alleles (SDAs) of SNP markers with a ratio of less than 2:1 among F_1_ progenies were used to construct a genetic linkage map as described by Li et al. [7]. SSR markers with a ratio of 1:1 in F_1_ progenies were used to construct genetic linkage maps. Those SDA markers and SSR markers were combined together to construct a genetic linkage map using JoinMap 4.0 [28]. First, markers were grouped using a minimum logarithm of odds (LOD) of five (> 5) for P1 and seven (> 7) for P2. Second, for each group, markers were clustered using default parameters. Linkage group numbers were assigned based on SSRs with known chromosome locations. The R package R/qtl was used to display the linkage map [33]. Phenotypic data were analyzed using means across the three replicates for each site/year and the BLUP values were used for QTL mapping. QTL IciMapping [34] was used for QTL analysis. A LOD threshold of three was used to identify potential QTL and the mapping method was inclusive composite interval mapping with additive effect (ICIM-ADD) in the BIP (biparental populations) functionality. The software MapChart [35] was used to display the QTL results.

## Abbreviations

Add: Estimated additive effect of the marker
BLUP: Best linear unbiased predictions
GBS: Genotyping by sequencing
GWAS: Genome-wide association studies
ICIM-ADD: Composite interval mapping with additive effect
LG: Linkage group
PVE: Phenotypic variation explained by QTL
QTL: Quantitative trait loci
RAD-seq: Restriction associated DNA sequencing
RFLP: Restriction fragment length polymorphism
SNP: Single nucleotide polymorphism
SSR: Simple sequence repeats
UNEAK: Universal network enable analysis kit
